# Sympatric, temporally isolated populations of the pine white butterfly *Neophasia menapia*, are morphologically and genetically differentiated

**DOI:** 10.1371/journal.pone.0176989

**Published:** 2017-05-31

**Authors:** Katherine L. Bell, Christopher A. Hamm, Arthur M. Shapiro, Chris C. Nice

**Affiliations:** 1 Biology Department, Texas State University, San Marcos, TX, United States of America; 2 Department of Ecology and Evolutionary Biology, University of Kansas, Lawrence, KS, United States of America; 3 Center for Population Biology, University of California, Davis, CA, United States of America; University of Innsbruck, AUSTRIA

## Abstract

Temporal isolation remains an understudied, and potentially under-appreciated, mechanism of reproductive isolation. Phenological differences have been discovered in populations of the pine white butterfly (*Neophasia menapia*), a typically univoltine species found throughout western North America. At two locations in the Coast Range of California there are two periods of adult emergence per year, one in early summer (July) and one in late summer/autumn (September/October). Differences in flight time are accompanied by differences in wing shape and pigmentation. Here we use a combination of population genomics and morphological analyses to assess the extent to which temporal isolation is able to limit gene flow between sympatric early and late flights. Not only did we detect both genetic and morphological differences between early and late flights at the two sites, we also found that the patterns of differentiation between the two flights were different at each location, suggesting an independent origin for the two sympatric flights. Additionally, we found no evidence that these sympatric flights originated via colonization from any of the other sampled localities. We discuss several potential hypotheses about the origin of these temporally isolated sympatric flights.

## Introduction

The study of the origin and maintenance of reproductive isolation remains a central focus in evolutionary biology and provides key insights into the process of speciation. Variation in phenology, the seasonal timing of life history events, can act as a reproductively isolating mechanism. Our knowledge of the evolutionary consequences of this isolation, specifically its role in diversification, is relatively incomplete [1–4]. Phenological differences may arise in response to other diversifying mechanisms. For example, environmental change, geographic isolation, or a shift in resource use may drive the evolution of phenology [5, 6]. In many cases temporal isolation is considered to reinforce reproductive isolation, rather than to be the primary isolating mechanism. The term allochronic speciation was developed to describe cases in which the initial stages of speciation are set in motion by a change in phenology [1, 7]. Once thought to be a relatively rare form of reproductive isolation, in recent years there have been examples of allochronic and temporal isolation across many diverse taxa; including insects [8–11], plants [12], birds [13], and corals [14], indicating that temporal differentiation is a potentially important isolating mechanism.

While temporal differentiation can facilitate divergence and speciation, regulation of activity and phenology typically results in synchronization of behavior within populations or species. Many factors may contribute to synchronization. For phenological synchronization in insects, one such strategy is diapause, a quiescent state in which annual periods of unfavorable climate are bypassed [15]. Shifts in phenology have been well documented, especially in insects, and often involve changes in diapause [16]. Diapause is wide spread among insects. It can occur at diverse life history stages, from eggs through to adults, but within a species it is typically restricted to a single stage [17]. Diapause is most frequently faculative, whereby the timing of diapause is mediated by environmental cues—such as day length [17]. When phenological shifts occur, presumably due to disruptive or divergent selection, synchronization within populations can reinforce divergence between populations. This temporal divergence can occur in sympatry, or in allopatry where it may be followed by range changes that bring the diverging populations into sympatry. We are interested in whether, and to what extent, these temporal life history changes restrict geneflow.

Here we investigate a possible case of temporal isolation in *Neophasia menapia*, the pine white butterfly, which occurs throughout western North America [15, 18]. The pine white is a univoltine species; adults emerge in summer, eggs are laid and overwinter (enter diapause) until the following spring when they hatch. Caterpillars feed on pine needles and develop directly, pupate, and adults emerge, mate and lay eggs that diapause the following winter [19–24]. In California, two locations in the Coast Range have been discovered where there are two periods of adult emergence per year, one in early summer (July) and one in late summer/autumn (September/October) (hereafter referred to as early and late flights respectively). At these two sites, differences in emergence time, early or late, appear to be accompanied by differences in wing morphology with the late flight appearing to have more melanization and broader wings than the early flight. The sympatric nature of these populations provides a novel opportunity to study changes in phenology without the confounding factor of geographic or habitat variation.

We use a combination of population genomics and morphological analyses to examine the extent to which these sympatric early and late flights in the Coast Range are differentiated and isolated and to test hypotheses on the possible origin of these sympatric flights. We address two specific questions: 1). Do sympatric early and late flights exhibit population genomic differentiation consistent with the hypothesis of temporal isolation? If no genetic differentiation is detected, this would be consistent with the alternative hypothesis that *N. menapia* at these sites comprise a single population that might have undergone a shift in life history to become bivoltine (two generations per year), or exhibits plastic variation for diapause emergence. If this is the case there would be no reproductive isolation as flight status would not be heritable. 2). How different are wing pigmentation and wing shape between the two sympatric flights at each of the sites, and compared to other nearby univoltine *N. menapia* populations? In addition, we explore what hypotheses can be proposed to explain the origin(s) of the sympatric populations. The sympatric populations can have arisen *in situ*, or one or both of the early and late flights could have originated elsewhere and colonized the Coast Range. A combination of high resolution, multi-locus genomic data and morphometric analyses was used to address these questions.

## Materials and methods

### Butterfly biology

The genus *Neophasia* (Pierinae) includes only two species worldwide, both occurring in North America. The common name of Pine White butterflies refers to their use of host plants from the Pinaceae [18], *Neophasia menapia* occurs throughout western North America while the second species, *Neophasia terlootii* occurs in southwestern USA and northwestern Mexico [18].

The wings of *N. menapia* are white with strong black markings around the leading edge of the forewing that curve around to form a cell-end bar [24, 25]. There are black markings along the veins of the hind wings in both males and females [26]. Many females have bright pinkish-red markings along the apical margin of the underside of the hind wing [26].

Throughout their range *N. menapia* are univoltine, meaning they have one generation and one flight period per year [18, 27–32]. The exception to this is at two locations, Goat Mountain and Mendocino Pass, in the Coast Range in California. At these locations there are two distinct flight periods and individuals from alternate flights (early vs. late) appear to exhibit morphological differences. The flight periods at these two sites largely spans the variation in flight period observed in univoltine single flight populations found throughout the species range. Univoltine populations of *N. menapia* are known to fly from late July until early September, and are most common in August [19–22]. This variation in the timing of flight period is represented in our study in the allopatric populations sampled from the Sierra Nevada in California, and from Oregon (Table 1). For these allopatric populations we use collection dates as a proxy for the timing of their flights, as flight windows in these populations of *N. menapia* have not been systematically examined. It has been suggested that elevation may affect the time of flight, with earlier flights (July) occurring at low elevations and later flights (September) occurring at high elevations [18, 30].

**Table 1:** Sampling locations for *Neophasia menapia* and *Neophasia terlootii* used in genomic analyses. Number in parentheses after each collection date represents the number of individuals collected in that year.

Species	Site Location	Abbreviation	Site Details	Latitude	Longitude	Elevation (ft.)	Sample SIze	Collection Date
*N. menapia*	Donner Pass, CA	DP	Sierra Nevada	39.31907	−120.3285	7,000	23	September ’95
	Lang Crossing, CA	LA	Sierra Nevada	39.31879	−120.6572	4,528	20	August ’95
	Woodfords, CA	WO	Sierra Nevada	38.77768	−119.8218	5,617	21	August ’95 (16), ’00 (5)
	Goat Mt. early CA	GE	Coast Range	39.26027	−122.7149	3,655	24	July ’95
	Goat Mt. late, CA	GL	Coast Range	39.26027	−122.7149	3,655	26	October ’95 (18), September ’99 (8)
	Mendocino Pass early, CA	ME	Coast Range	39.79432	−122.9350	5,000	26	July ’95 (15), ’00 (11)
	Mendocino Pass late, CA	ML	Coast Range	39.79432	−122.9350	5,000	20	September ’95 (18), ’99 (2)
	Otis, OR	OR	Coast Range	45.02440	−123.9453	46	12	September ’00
*N. terlootii*	Cochise County, AZ	AZ	Huachuca Mt.s			9,500	14	October ’91 (4), November ’02 (6), ’04 (4)

Females lay eggs in rows along pine needles in groups of up to 40, they overwinter (diapause) as eggs, and larvae begin feeding in spring [18, 30]. In both the Sierra Nevada and the Coast Range their host plant, Ponderosa Pine (*Pinus pondersoa*) is ubiquitous. While there are no mark-recapture studies of *Neophasia* to allow direct estimates of dispersal, our personal observation is that they are weak fliers with “strays” very rarely being encountered. Movements appear to be primarily between nectar sources and lekking sites, which are tall trees (usually the host).

To the best of our knowledge, *N. menapia* is not known to exhibit wing pattern polyphenism (seasonal or otherwise), nor is there any evidence of multiple generations. Unfortunately, females fail to oviposit in laboratory settings (A.M. Shapiro, pers. obs.) which prevents manipulative experimental approaches to investigating the mechanisms of phenotypic differentiation. Therefore, we have approached the study of differentiation from a geographical, comparative perspective.

### Sampling and collection

**Fig 1:**
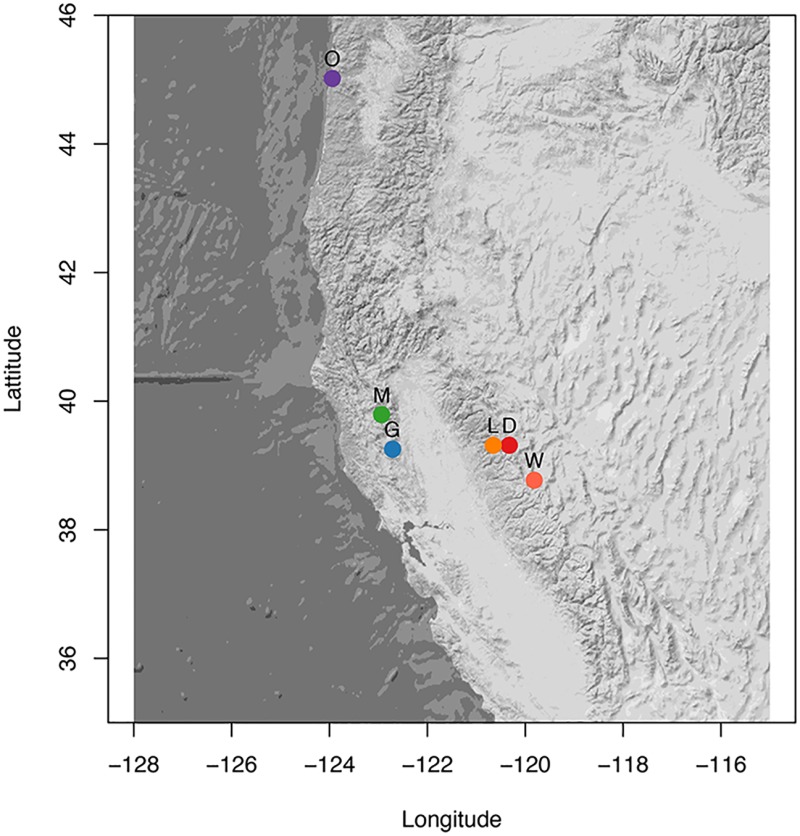
Map of sampling locations. Map of *N. menapia* sampling locations; D (red) = Donner Pass, L (orange) = Lang, W (light red) = Woodfords, G (dark blue) = Goat Mounain, M (dark green) = Mendocino Pass late flight, O (purple) = Oregon. *N. terlootii* were sampled from Arizona, not shown on the map.

A total of 187 butterflies were collected between 1995 and 2004 at several locations across California, Arizona and Oregon (Table 1). No permits were required for the collection of *Neophasia* at these locations. Neither of the two species of *Neophasia* are listed as endangered or protected. We collected 173 *N. menapia* at five sites in California, and one site in Oregon (Fig 1). At both Goat Mountain and Mendocino Pass in the Coast Range, two flights, early and late, have been observed. At these sites individuals were collected during both periods of adult flight, resulting in an early and a late group for both sampling locations. The extent to which these two flights are locally sympatric is not clear, thus it is uncertain what role environmental factors play in determining phenological differences. The late flights at both Goat Mountain and Mendocino Pass seem to be more associated with west-facing slopes, whereas the early flights are more commonly collected on east-facing aspects. Individuals at each flight have been collected in close proximity, albeit at very different times, and the butterflies are certainly capable of flying across the entire area where the two flights are encountered. We consider the early and late flights at Goat Mountain and Mendocino Pass to be broadly sympatric. Beyond the Coast Range, three sites in the Sierra Nevada were sampled: Lang Crossing, Woodfords and Donner Pass and one site in Oregon (Fig 1). All locations sampled in the Sierra Nevada CA and Oregon were univoltine (one generation/flight per year). In Arizona 14 *N. terlootii*, the only other species in the genus, were sampled and included as a basis for comparison in the analysis of population structure of *N. menapia*. All samples were kept at −80°C until DNA extraction.

### Molecular methods

Next generation DNA sequence data were generated following Gompert et al. (2012) and Parchman et al. (2012) [33, 34]. DNA was isolated and purified from each sampled butterfly from approximately 0.1 grams of thoracic tissue using: (i) QIAgen’s DNeasy 250 Blood and Tissue Kit (QIAgen Inc.) in accordance with the manufacturer’s protocol or (ii) standard phenol-chloroform protocol [35]. We fragmented DNA using two restriction enzymes (EcoR1 and Mse1) resulting in a genomic DNA library for each individual. Customized Illumina adaptor sequences and an eight to ten base pair MID (multiplex identifier) barcode were ligated to DNA fragments for each individual. Two rounds of PCR were used to amplify individual libraries, after which PCR products were pooled across all individuals. This resulted in a pooled library for 187 individuals, with fragments identifiable by unique 8–10bp barcodes. Pooled PCR products were separated on a two percent agarose gel and fragments between 300–500bp were selected by purifying them from the gel using QIAquick gel extraction kit (QIAgen Inc.) as per the manufacturer’s protocol. These reduced representation genomic libraries were sequenced at the National Center for Genomic Research (Santa Fe, NM) using Illumina HiSeq version 2 chemistry.

We obtained 36 million sequence reads which were processed using a series of quality control steps to identify variable sites, following the methods of Gompert et al. (2012) [33]. In overview, custom perl scripts were used to identify sequences to an individual based on barcode sequences. We then removed barcodes and removed sequences that contained adaptor sequence, or that were of poor quality. De novo assembly was conducted on a subset of reads (11.2 million) using Seqman Ngen 3.0.4 (DNASTAR). Consensus sequences from the assembly were concatenated to produce an artificial chromosome for reference-based assembly of the total 36 million reads using Seqman Ngen 3.0.4 (DNASTAR). Variable sites were called using custom Perl scripts, SAMtools and bcftools [36]. A minimum of 25 percent coverage at a site was required for the site to be called as variable. We assumed an infinite sites model, thus all variable sites with more than two nucleotides (alleles) were removed. This resulted in 40,389 variable sites. To quantify genetic variation across the genome for each population we used SAMtools and bcftools [36] to obtain estimates of *π*, the expected herterozygosity, and Watterson’s *θ* for each population. To obtain the estimates we used expectation-maximization (EM) algorithm with 20 iterations for each populations [37].

### Population genetic analyses

Data were trimmed to only include Single Nucleotide Polymorphisms (SNPs) with a minimum of 15 reads per population sample, producing 20,737 SNPs. We used the allele frequency model presented in Gompert and Buerkle (2011) [38] to estimate allele frequencies for each locus based on the observed data; this is a similar approach to that used by Pritchard et al. (2000), Gillespie (2004), and Hedrick (2005) [39–41]. The model treats genotypes and allele frequencies as parameters that are estimated from the sequence data. For a more detailed description see Gompert and Buerkle (2011), and Parchman et al. (2012) [34, 38]. The posterior probabilities of parameter estimates (allele frequencies per population and genotype probabilities per locus per individual) were obtained using Markov Chain Monte Carlo (MCMC) with 100,000 steps and a burn-in of 10,000.

Genetic structure at the individual level was summarized using a principal component analysis (PCA) and the admixture model in STRUCTURE 2.3.4 [39, 42]. The PCA was conducted using genotype posterior probabilities for the 3 genotypes at each SNP (20,737) for each individual, using the statistical program R (using the prcomp function in the composition package in R). We produced two PCA’s, one that includes both nominal species, *N. terlootii* and *N. menapia*, and a second PCA using only *N. menapia* populations. For the analysis using the program STRUCTURE, we sampled one sequence read for each SNP locus for each individual in proportion to the frequency of reads at that locus for each individual. Thus individuals were assigned either a 1 or a 2 depending on which sequence read was sampled for that individual and −9 (missing data) for the alternative allele for each locus (script written by T. Parchman, University of Nevada, Reno). Our infile is similar to that used for dominant markers where heterozygosity at a locus cannot be verified. Individuals with more than 98 percent missing data were removed (1 individual from *N. terlootii* population, 4 individuals from Goat Mountain late population sample). For the STRUCTURE analysis 19,152 SNPs were included. The admixture model was used to estimate admixture proportions of each of K groups. Again, two analyses were conducted, one that included both nominal species and one that included just *N. menapia* populations. The model was run for K = 1–12 and K = 1–10 respectively, with 10 runs per K. Monte Carlo Markov Chain (MCMC) procedures were used to obtain estimates, with 100,000 steps and a burn in of 50,000 steps. To estimate the appropriate K (number of groups) the log of the marginal likelihood [39] was plotted against K and the ad hoc Δ K statistic was calculated and plotted against K [43]. At the population level we calculated pairwise G_*ST*_ statistics among all populations from allele frequency estimators [44]. G_*ST*_ estimates were summarized using a non-metric multidimensional scaling (NMDS) conducted in R using the package MASS.

### Geometric morphometrics

To quantify variation in wing pigment patterns (melanization) and wing shape, forewings of male *N. menapia* were photographed using a digital camera (Sony Cyber-shot HX9V) on a white background with a scale (mm ruler) (Table A in S1 File). As our sample included more males than females, we used only male wings in order to avoid complications from sexual dimorphism. Measurements were taken for the left forewing unless there was wing damage, in which case, the right wing was used. Specific damage to a wing could lead to the exclusion of that sample from either the wing pigment analysis or the wing shape analysis, leading to differing samples sizes between the two approaches.

#### Wing melanization

All measurements for wing melanization were taken using IMAGEJ software [45]. The area of each wing was measured twice and the average of the two measurements was used in all analyses. Images were transformed to grey scale and then made binary, allowing the total area of black on the wing to be measured. Any white that was within black areas was selected and total melanization was calculated as black area minus white area. Each measurement was taken twice and the average of the two was used in calculations. We calculated the correlation coefficient between melanization and wing area and found there to be a significant positive correlation (R = 0.3169216, P<0.001), we therefore conducted a regression of total melanization on wing area in order to control for differences in wing size. The regression of total melanization on wing area was conducted using the function glm in R [46], and the residuals used in further statistical analysis. A one-way ANOVA followed by Tukey’s HSD was used to examine which populations differed significantly in wing melanization [46].

#### Wing shape

We identified 12 landmarks, located either at convergence points between wing veins or the intersection of a vein and the edge of the wing (Fig A in S1 File). X Y, co-ordinates of the landmarks were measured using IMAGEJ software. Co-ordinates were imported into MorphoJ for further analyses [47]. A generalized procrustes analysis, which removes non-shape variation such as rotation and scale, was used to normalize co-ordinates [48]. In order to control for allometry (variation in shape because of size), a multivariate regression of wing shape (dependent variable) on centroid size (independent variable) was conducted in MorphoJ software [47]. Centroid size is an isometric estimator of size calculated by taking the square root of each summed square distance of each landmark from the center of the landmark configuration [49]. The residuals of this regression were used in all subsequent analyses. To identify the main axes of variation within the data set, we conducted a principal component analysis, using a covariance matrix in MorphoJ. We then carried out three ANOVA’s, one using PC1 scores, a second using PC2 scores and finally one with PC3 scores. A Tukey’s HSD post hoc test was then used to examine which pairwise comparisons were significantly different. We also used a canonical variate analysis (CVA) to explore patterns of variation among groups. In this analysis groups are identified *a priori* and canonical variables are calculated that maximize the amount of among group variance relative to within groups. This allows for visualization of the variation among groups. For both the PCA and the CVA, 95% confidence ellipses around the mean, using population as a classifier, were plotted. For CV1 and CV2 a transformation grid plot showing wing shape changes was plotted in MorphoJ [47].

## Results

### Population genetics

**Fig 2:**
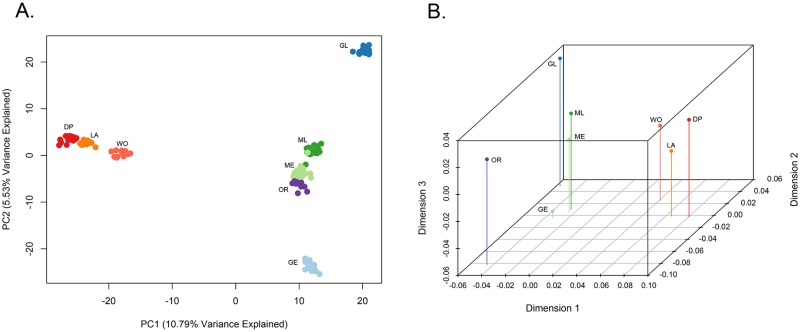
Principal component analysis of genotype posterior probabilities and non-metric multi-dimensional scaling graph of pairwise G_*ST*_. A: PCA for *N. menapia* population samples based on genotype posterior probabilities where each circle represents an individual’s genotype posterior probabilities across all 20,737 SNPs; B: Non-metric Multi-dimensional Scaling (NMDS) graph of pairwise G_*ST*_ estimates among populations of *N. menapia*, showing 3 dimensions; DP (red) = Donner Pass, LA (orange) = Lang, WO (light red) = Woodfords, GE (light blue) = Goat Mountain early flight, GL (dark blue) = Goat Mountain late flight, ME (light green) = Mendocino Pass early flight, ML (dark green) = Mendocino Pass late flight, OR (purple) = Oregon.

We used approximately 20,000 SNPs (20,737 SNPs for PCA and G_*ST*_, 19,152 SNPs for STRUCTURE analysis) obtained from assembly of 36 million Illumina sequence reads. A principal component analysis (PCA) was conducted on all eight *N. menapia* sample groups and the one group of *N. terlootii* (Fig B in S1 File). PC1 explained 26.04% of the variance and divided groups based on their nominal species designation. *N. terlootii* is clearly distinguished from all *N. menapia* populations. PC2, which explained 7.9% of the variance, showed subdivision among the *N. menapia* population samples, with Coast Range populations (Goat Mountain early and late, Mendocino Pass early and late and Oregon) clustering together, separate from Sierra Nevada sites (Donner Pass, Lang and Woodfords). A second PCA was conducted to explore patterns of differentiation among the *N. menapia* samples (Fig 2A). PC1, which explained 10.79% of the variance, separated Coast Range and Sierra Nevada samples while PC2, which explained 5.53% of the variance, showed further subdivision within the Coast Range populations. Sympatric early and late flights at Goat Mountain clustered separately, at opposite ends of PC2 axis. Mendocino Pass early and late flights did not show the same level of genetic differentiation and were closer together towards the center of PC2. The Oregon population clustered close to Mendocino Pass early and late flight populations.

All pairwise comparisons resulted in G_*ST*_ values significantly different from zero (Table 2). Pairwise G_*ST*_ comparisons between each *N. meanpia* sampling location and *N. terlootii* were of a similar scale and higher than any of the intraspecific comparisons. G_*ST*_ between early and late flights at Goat Mountain was similar to G_*ST*_ between Goat Mountain and other, geographically isolated populations. At Mendocino Pass, G_*ST*_ between early and late flights was significantly different from zero but was relatively low compared to other G_*ST*_’s. A non-parametric multi-dimensional scaling analysis (NMDS) was used to visualize the relationships between *N. menapia* sampling groups using pairwise G_*ST*_ values and showed patterns of relatedness (Fig 2B) similar to those seen in the PCA plots based on the individual genotype probabilities. The three Sierra Nevada sites clustered together (Donner Pass, Lang and Woodfords). Mendocino Pass early and late populations clustered relatively close together while the early and late flights at Goat Mountain clustered at opposite ends of dimension three reflecting genetic differentiation between early and late flights at this site. The Oregon sample is distinct, but remains closer to the Californian Coast Range populations relative to the Sierra Nevada sites.

**Table 2:** Pairwise *G*_*ST*_’s calculated from allele frequencies: Lower triangle *G*_*ST*_ estimate, top triangle 95% credible intervals.

	AZ	DP	LA	WO	GE	GL	ME	ML	OR
AZ		0.449–0.456	0.446–0.452	0.442–0.449	0.446–0.453	0.451–0.458	0.439–0.446	0.440–0.447	0.447–0.455
DP	0.452		0.032–0.034	0.039–0.040	0.064–0.065	0.071–0.073	0.055–0.056	0.058–0.060	0.075–0.077
LA	0.448	0.033		0.054–0.055	0.059–0.060	0.066–0.068	0.050–0.051	0.053–0.054	0.071–0.072
WO	0.449	0.040	0.035		0.054–0.055	0.062–0.063	0.044–0.046	0.048–0.049	0.066–0.068
GE	0.449	0.064	0.060	0.054		0.056–0.057	0.038–0.039	0.044–0.041	0.061–0.063
GL	0.454	0.072	0.067	0.063	0.057		0.043–0.044	0.041–0.043	0.066–0.068
ME	0.442	0.055	0.051	0.045	0.038	0.043		0.030–0.031	0.052–0.054
ML	0.443	0.059	0.054	0.049	0.044	0.042	0.031		0.054–0.055
OR	0.451	0.076	0.071	0.067	0.062	0.067	0.053	0.055	

**Fig 3:**
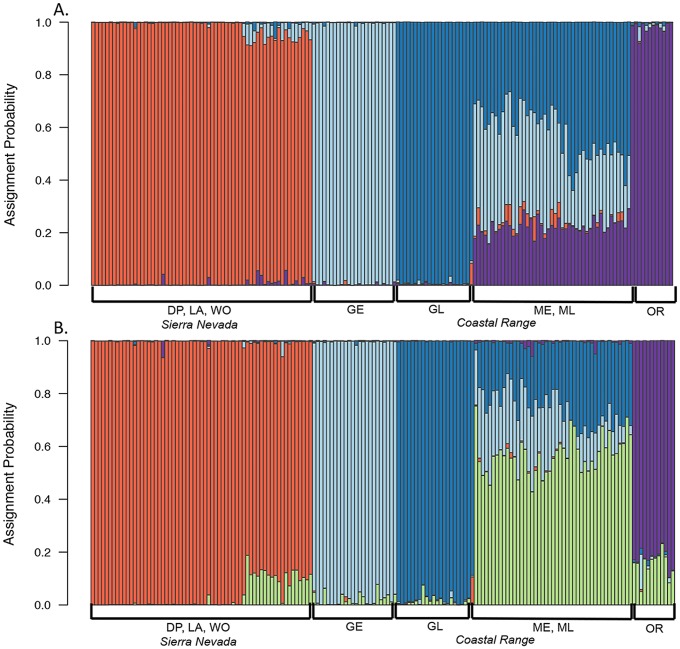
Structure plot for *N. menapia* populations. STRUCTURE assignment plots for all 8 *N. menapia* population samples. A: Assignment probabilities from STRUCTURE for K = 4, orange = Sierra Nevada populations, light blue = GE, dark blue = GL, purple = Oregon. B: Assignment probabilities for K = 5, orange = Sierra Nevada populations, light blue = GE, dark blue = GL, green = Mendocino Pass, purple = Oregon. DP = Donner Pass, LA = Lang, WO = Woodfords, GE = Goat Mountain early flight, GL = Goat Mountain late flight, ME = Mendocino Pass early flight, ML = Mendocino Pass late flight, OR = Oregon.

In the first STRUCTURE analysis that included both species, K = 2 or K = 3 were found to be the best clustering solutions. When assignment probabilities were plotted for K = 2, *N. terlootii* formed one cluster, while the *N. menapia* samples formed a second cluster (Fig C S1 File). For K = 3, *N. terlootii* formed the first cluster, then *N. menapia* populations split into two clusters, populations from the Coast Range and populations from Sierra Nevada (Fig C S1 File). For *N. menapia*, K was found to be either 4 or 5 (Fig D S1 File). When assignment probabilities for K = 4 were plotted the three Sierra Nevada sites group together, early and late flights at Goat Mountain formed two separate clusters, early and late flights at Mendocino Pass formed an apparently admixed group and the Oregon sample formed its own cluster but with some assignment to the Mendocino Pass cluster (Fig 3). For K = 5, the groups stay the same but Oregon forms its own cluster, distinct from the two Mendocino groups (Fig 3).

Genetic diversity estimates for all *N. menapia* populations are shown in Fig E in S1 File. Genetic diversity was found to be similar across all populations. Neither the early or late flights at Goat Mountain and Mendocino Pass have exceptional amounts of variation, and variation across all populations is similar to that observed for other butterflies [50].

### Geometric morphometrics

#### Wing melanization

**Fig 4:**
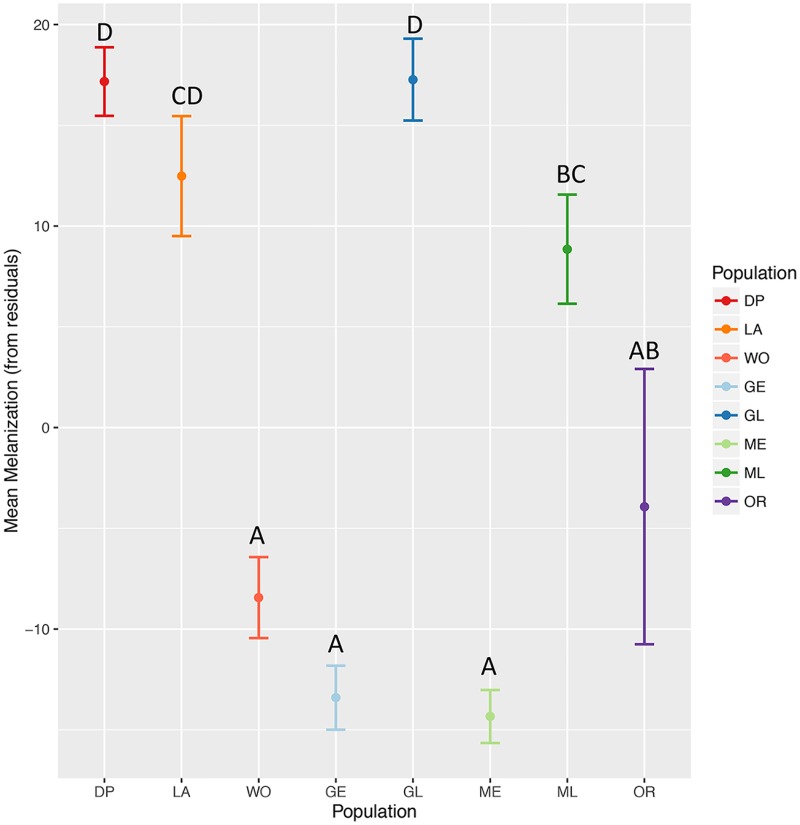
Mean melanization for *N. menapia*. Mean melanization, including error bars, for each *N. menapia* population sample, unique letters indicate significant differences in melanization (calculated using Tukeys HSD post hoc test). DP = Donner Pass, LA = Lang, WO = Woodfords, GE = Goat Mountain early flight, GL = Goat Mountain late flight, ME = Mendocino Pass early flight, ML = Mendocino Pass late flight, OR = Oregon.

Mean values of melanization (from residuals; plus or minus standard error) were plotted for each sampling group (Fig 4). Goat Mountain early flight and Mendocino Pass early flight have very similar mean levels of melanization. The next closest group is Woodfords and then Oregon. Furthest from the two early flights are Donner Pass and Goat Mountain late flight; these groups have similar mean melanization. With approximately intermediate levels of melanization are Lang and Mendocino Pass late flight. A one-way ANOVA was conducted to explore variation in melanization between populations. Significant differences in melanization per population were found (*F*_7,188_ = 41.12, P< 2e-167). A post hoc test, Tukey’s HSD test, was carried out to identify which pairwise comparisons were significantly different (Table B in S1 File). Differences were found between sympatric early and late flights at both Goat Mountain and Mendocino Pass. Several other pairwise comparisons showed significant differences in melanization. Non-significant differences were found in 11 pairwise comparisons (out of 28).

#### Wing shape

**Fig 5:**
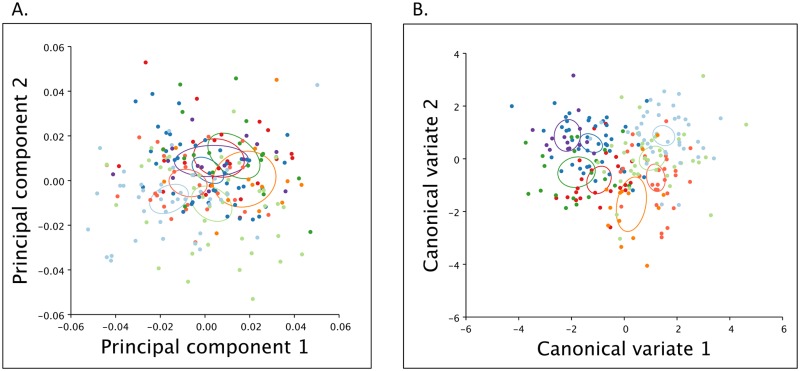
Principal component analysis and canonical variance analysis of wing landmarks. A: PCA of *N. menapia* wing landmarks, 95% confidences ellipses around the mean for each population. B: CVA of *N. menapia* wing landmarks, 95% confidence ellipses around the mean for each population. *N. menapia* samples from; DP (red) = Donner Pass, LA (orange) = Lang, WO (light red) = Woodfords, GE (light blue) = Goat Mountain early flight, GL (dark blue) = Goat Mountain late flight, ME (light green) = Mendocino Pass early flight, ML (dark green) = Mendocino Pass late flight, OR (purple) = Oregon.

A PCA was carried out on the 12 landmarks to identify the main axes of variation in wing shape. When PC1 (24.45% variance explained) and PC2 (15.48% variance explained) are plotted there appears to be little discernible clustering by sampling group (Fig 5A). 95% confidence ellipses around the mean for each population sample show overlap between several populations but not between the early and late flights at either Goat Mountain or Mendocino Pass. To test statistically for differences between groups and their PC scores, a one way ANOVA was used with Tukey’s HSD post hoc test to identify which pairwise comparisons were significantly different (Table D in S1 File through Table I in S1 File). For both PC1 and PC2 scores, significant differences were found between early and late flights at Goat Mountain, but not for PC3. At Mendocino Pass there were significant differences between early and late flights for their PC2 scores.

To further explore patterns of variation of wing shape among groups, a CVA was used. CVA differs from a PCA because groups are assigned *a priori* and the analysis maximizes among-group differences relative to within-group differences. In a plot of CV1 (47.65% variance explained) and CV2 (16.07% variance explained), Goat Mountain early flight sample clusters towards the far end of CV1 away from the late flight at Goat Mountain, the same pattern can be seen for Mendocino Pass early and late flight groups (Fig 5B). The 95% confidence ellipses demonstrate differences in the mean between early and late flights at both sites. The three Sierra Nevada populations cluster relatively close together but do not overlap. Transformation grid plots show that for CV1 there are noticeable shifts in landmark 1 and landmark 7 as well as slight changes in several other landmarks. For CV2 there are also changes in landmarks 1 and 7 as well as changes in landmark 8 (Fig F S1 File). As was found with wing melanization, differences in wing shape were found between early and late flights, but this variation falls within the variation seen between other sampling locations.

## Discussion

We used a genome-wide survey of DNA sequence variation and morphological analyses of wing shape and pigmentation to explore the evolutionary significance of sympatric early and late flights of *N. menapia* at two locations in California. Our data were used to test the hypothesis of temporal isolation between sympatric early and late flights and examine various hypotheses about their origin. We found strong genetic differentiation between the two nominal species, *N. terlootii* and *N. menapia*. Within *N. menapia* we found significant genetic and morphological differences between sympatric early and late flights at both sites in the California Coast Range. Interestingly, patterns of genetic differentiation were variable among the two sites, with Goat Mountain early and late flights showing higher levels of differentiation than early and late fights at Mendocino Pass. Patterns in wing morphology were also variable between the two sites. However, patterns of genetic structure and morphological structure are not congruent. We found no evidence that the flights originated from an allopatric population in the Sierra Nevada. In the PCA, NMDS or STRUCTURE analyses, the Coast late flight populations never clustered with Sierra Nevada populations and were closer to the early flight populations, we therefore conclude that they either originated from within the Coast Range, or from an un-sampled allopatric population (Figs 2 & 3).

To return to our initial research questions: we first wanted to explore the population genomics of sympatric early and late flights and identify if there were levels of genetic structure present that would be consistent with the hypothesis of temporal isolation. Our results provide support for the hypothesis of temporal isolation between sympatric early and late flights at both locations. At Goat Mountain, populations show higher levels of genetic differentiation relative to Mendocino Pass, as can be seen in the PCA of individual genotypes and the NMDS of pairwise G_*ST*_’s (Fig 2). At Goat Mountain differentiation between early and late populations is at a similar scale to differentiation between geographically isolated populations located in different mountain ranges (Sierra Nevada vs. Coast Range) (Table 2). At Mendocino Pass differentiation was not as great as that observed at Goat Mountain, but the *G*_*ST*_’s calculated between early and late flights was significantly different from zero. This provides strong evidence against the hypothesis that *N. menapia* populations have switched from a univoltine (one generation per year) to a bivoltine (two generations per year) life cycle, or that emergence date is a non-heritable plastic trait. If populations had become bivoltine or emergence date was plastic then we would not expect to identify any significant genetic differentiation as early flight individuals would represent the parental populations of late flight individuals (and vice versa).

Our second question asked if sympatric early and late flights differed from each other, and other allopatrically isolated *N. menapia* populations in wing pigmentation (melanization) and wing shape. These morphological traits were chosen based on field observations and represent a preliminary assessment of potentially adaptive differences between early and late flights. Significant differences in both wing melanization and wing shape were found between sympatric early and late flights at both Goat Mountain and Mendocino Pass (Figs 4 & 5). An ANOVA found significant differences in melanization between early and late flights at both sites, as well as between pairwise comparisons of several other allopatric sites (Fig 4). For wing shape we found several significant differences between populations using an ANOVA. As with melanization, there were differences between early and late flights, and among several other comparisons. Patterns of wing shape differentiation did not reflect either the patterns seen in melanization or the genetic patterns identified. The CV_1_ axis appears to divide early vs. late flights, while CV_2_ divides populations based on sampling location (Fig 5). We found no overlap in the 95% confidence ellipses of the mean, for early and late flight populations (Fig 5). The mechanism underlying variation in melanization and wing shape in this species remains unknown. Increased melanization on the distal portion of the forewing is unlikely to play a thermoregulatory role, and to our knowledge there is no evidence of wing polyphenism in this species [51]. However, it is certainly possible that the morphological differentiation among the sampled populations is attributable to plasticity in response to environmental differences, at least in part. This research does not address the likelihood that morphological differences are the result of plasticity or genetic changes, but aims to take the initial step of quantifying differences. Regardless of the underlying basis of wing morphology in this species there is the potential that the observed morphological patterns could represent adaptive evolutionary change [52]. Further research would be required to assess the underlying basis of these traits, and the possible evolutionary significance of this variation.

Our final question aimed to explore hypotheses about the possible origins of sympatric early and late flights. The genetic differentiation seen among *N. menapia* populations is not at the same scale as that between *N. menapia* and its sister species *N. terlootii*. This indicates that isolation between *N. menapia* populations is relatively recent and/or there is ongoing gene flow to some extent among *N. menapia*. Two alternate hypotheses about the origin of early and late flights involve either colonization occurring from one (or more) Sierra Nevada sites or that sympatric flights have arisen from within the Coast Range. We have found no support for the first hypothesis, colonization from an allopatric Sierra Nevada population. In terms of genetic differentiation the NMDS plot, PCA, and STRUCTURE assignment probability plots (Figs 2 & 3) demonstrate that there is clear differentiation between populations from the Sierra Nevada and Coast Range. This includes Oregon clustering with Coast Range sites in California despite considerable geographic distance, indicating that gene flow within ranges is more likely than between the Coast Range and the Sierra Nevada. Further geographic sampling is required to identify areas in the Coast Range that could be the source of colonists to either the early or late flight. We know of no other localities with sympatric, phenologically isolated flights of *N. menapia*, however, Shapiro et al. (1979) [53] noted phenological differences between populations of *N. menapia* in the Trinity Alps in northwestern California. There, butterflies at lower elevations (900m) fly earlier (June–July) and higher elevation (1500m) butterflies appear later (September–October), but without the phenotypic differentiation observed at Goat Mountain and Mendocino Pass. Similar phenological isolation has also been noted for other species of butterflies. For example, Shapiro and Forister (2005) [54] described phenologically isolated populations of skippers in the *Hesperia colorado* complex, with the later-flying population at one sympatric site being associated with serpentine soils. However, the causes of phenological isolation in that case, as with *N. menapia*, remain mysterious. Furthermore, we are not presently able to identify if sympatric early and late populations of *N. menapia* arose *in situ* or if there has been a colonization event from another Coast Range population that was not included in our sampling.

Although population genetic differentiation has been identified between sympatric populations at both sites, the extent of differentiation is not the same. Variation between the two sites could indicate that the process of temporal isolation is variable. For example, the origin of temporal isolation could be different; i.e. at one site a temporally isolated population has arisen *in situ*, while at the other site colonization from an allopatric population with a later flight time may have occurred. Alternatively it may be that the two sites are different because isolation has arisen in sympatry at different times; Goat mountain populations may have been isolated from one another for longer than those at Mendocino Pass. Morphological measurements, wing melanization and wing shape (Figs 4 & 5), do not reflect these genetic patterns and are not consistent with one another in terms of structure among populations. Given that the genetic basis of these traits is unknown for this species, it would be inappropriate to infer evolutionary relationships based on these data.

In order to explore these unanswered questions, and other evolutionary details of these temporally isolated sympatric populations, further research is required. For example, further geographical sampling, lab-based experiments to examine variation in the dynamics and control of diapause, especially the termination of diapause, or exploration of the potential adaptive significance of wing morphology would expand our understanding of the evolutionary significance of this temporal isolation.

In conclusion, this study has investigated two cases of temporal isolation in the pine-white butterfly, suggesting that it is an important isolating mechanism for this species. Both genetic differentiation and morphological differences were found between sympatric early and late flights at the two sites. We determine the biogeographic origin of populations at the sympatric sites is likely to have come from within the Coast Range, not from the Sierra Nevada. This case, along with other recent work on temporal isolation [1, 9–11, 13, 55, 56] demonstrates that temporal isolation may occur more frequently than previously thought and warrants further research into the underlying mechanism of this process of reproductive isolation.

## Supporting information

S1 File Supporting information. **Fig A**, **Location of Landmarks**. Left panel: male forewing from Goat Mountain early flight. Middle panel: location of 12 landmarks on *N. menapia* forewing, wing changed to greyscale in ImageJ. Right panel: Male forewing from Goat Mountain late flight. **Fig B**, **Principal Component Analysis of Genotype Posterior Probabilities**. PCA based on genotype posterior probabilities where each circle represents an individual’s genotype posterior probabilities across all 20,737 SNPs; PCA for *N. terlootii* and *N. menapia*. AZ (yellow) = *N. terlootii* from Arizonia, *N. menapia* samples from; DP (red) = Donner Pass, LA (orange) = Lang, WO (light red) = Woodfords, GE (light blue) = Goat Mountain early flight, GL (dark blue) = Goat Mountain late flight, ME (light green) = Mendocino Pass early flight, ML (dark green) = Mendocino Pass late flight, OR (purple) = Oregon. **Fig C**, **Structure Plot for All Populations**. A: STRUCTURE assignment plot for K = 2, includes all populations samples (*N. terlootii* and *N. menapia*); dark blue = AZ (*N. terlootii*), medium blue = all *N. menapia* populations. B: STRUCTURE assignment plot for K = 3, includes all populations samples (*N. terlootii* and *N. menapia*), dark blue = AZ, light blue = Sierra Nevada *N. menapia*, medium blue = Coast Range *N. menpia*. AZ = *N. terlootii*, DP = Donner Pass, GE = Goat Mountain early flight, GL = Goat Mountain late flight, LA = Lang, ME = Mendocino Pass early flight, ML = Mendocino Pass late flight, OR = Oregon, WO = Woodfords. **Fig D**, **Delta K for K 2 through K 10 for *N. menapia* STRUCTURE Runs**. **Fig E**, **Genetic Diversity Estimates for *N. menapia*** Bars show estimates of heterozygosity (*π*), square shows the estimate of Watterson’s *θ*. **Fig F**, **Transformation Grid for Landmarks**. Transformation grid for landmarks from CV1 (top) and CV2 (lower). **Fig G**, **Boxplots of melanization level for populations of *Neophasia menapia***. Unique letters indicate significant differences in melanization (calculated by Procrusted distance ANOVA.) DP = Donner Pass, GE = Goat Mountain early flight, GL = goat Mountain late flight, LA = Lang, ME = Mendocino Pass early flight, ML = Mendocino Pass late flight, OR = Oregon, WO = Woodfords. **Table A**, **Sample sizes for *Neophasia menapia* for wing melanization and wing shape**. **Table B**, **Tukey’s HSD test for wing melanization**. Significant differences are highlighted in bold. **Table C**, **Pairwise Procrustes distances among populations for shape (upper triangle)**, **and melanization level (lower triangle)**. *P≤0.05, **P≤0.01, **P≤0.001. Table D, One-way ANOVA of wing shape PC1 by sampling location. Table E, One-way ANOVA of wing shape PC2 by sampling location. Table F, One-way ANOVA of wing shape PC3 by sampling location. Table G, Tukey’s HSD test for PC1 of wing shape. Significant differences highlighted in bold. Table H, Tukey’s HSD test for PC2 of wing shape. Significant differences highlighted in bold. Table I, Tukey’s HSD test for PC3 of wing shape. Significant differences highlighted in bold.
(PDF)
