# Supplementary material for: Sympatric, temporally isolated populations of the pine white butterfly Neophasia menapia, are morphologically and genetically differentiated
Source: PLoS One. 2017 May 31;12(5):e0176989. doi: 10.1371/journal.pone.0176989 (PMC5451007; doi:10.1371/journal.pone.0176989)
Supplement: S1 File — Fig A, Location of Landmarks. Left panel: male forewing from Goat Mountain early flight. Middle panel: location of 12 landmarks on N. menapia forewing, wing changed to greyscale in ImageJ. Right panel: Male forewing from Goat Mountain late flight. Fig B, Principal Component Analysis of Genotype Posterior Probabilities. PCA based on genotype posterior probabilities where each circle represents an individual’s genotype posterior probabilities across all 20,737 SNPs; PCA for N. terlootii and N. menapia. AZ (yellow) = N. terlootii from Arizonia, N. menapia samples from; DP (red) = Donner Pass, LA (orange) = Lang, WO (light red) = Woodfords, GE (light blue) = Goat Mountain early flight, GL (dark blue) = Goat Mountain late flight, ME (light green) = Mendocino Pass early flight, ML (dark green) = Mendocino Pass late flight, OR (purple) = Oregon. Fig C, Structure Plot for All Populations. A: STRUCTURE assignment plot for K = 2, includes all populations samples (N. terlootii and N. menapia); dark blue = AZ (N. terlootii), medium blue = all N. menapia populations. B: STRUCTURE assignment plot for K = 3, includes all populations samples (N. terlootii and N. menapia), dark blue = AZ, light blue = Sierra Nevada N. menapia, medium blue = Coast Range N. menpia. AZ = N. terlootii, DP = Donner Pass, GE = Goat Mountain early flight, GL = Goat Mountain late flight, LA = Lang, ME = Mendocino Pass early flight, ML = Mendocino Pass late flight, OR = Oregon, WO = Woodfords. Fig D, Delta K for K 2 through K 10 for N. menapia STRUCTURE Runs. Fig E, Genetic Diversity Estimates for N. menapia Bars show estimates of heterozygosity (π), square shows the estimate of Watterson’s θ. Fig F, Transformation Grid for Landmarks. Transformation grid for landmarks from CV1 (top) and CV2 (lower). Fig G, Boxplots of melanization level for populations of Neophasia menapia. Unique letters indicate significant differences in melanization (calculated by Procrusted distance ANOVA.) DP = Donner Pass, GE = Goat M [file pone.0176989.s001.pdf]

Supplementary Material

Wing Melanization

To measure melanization, images were transformed to grey scale and then made binary, allowing the total area of black on the wing to be measured. Any white that was within black areas was excluded and total melanization was calculated as black area minus white area. Each measurement was taken twice and the average of the two was used in calculations. We used an advanced procrustes ANOVA (ap-ANOVA) (Collyer et al., 2014) in the GEOMORPH package to quantify the amount of forewing melanization that was accounted for by population. For this analysis, we first fit a reduced model (intercept only) and then a second ap-ANOVA with the population parameter. The two nested models were then compared with 10,000 iterations of the residual randomized permutation procedure (RRPP), which allows generation of empirical P values and pairwise Procrustes distances between all groups (Collyer et al., 2014).

Wing Melanization

Using np-MANOVA, we observed that levels of melanization varied significantly among most populations of *N. menapia* ($F_{7,172}$ 42.55 $P < 9.9e^{-5}$) (Figure F; Supplemental Table 1). We found that Mendocino early and late populations were significantly different for melanization levels (Procrustes distance 22.7, $P < 0.001$), as were Goat Mountain early and late populations (Procrustes distance = 34.9, $P < 0.001$); several other comparisons showed significant differences in melanization. The early flights from Goat Mountain and Mendocino Pass possessed similar levels of melanization, which were lower than most other *N. menapia* populations. The late flights at Goat Mountain and Mendocino Pass had significantly higher levels of melanization than the early flights at those sites, but Goat Mountain possessed higher levels of melanization than Mendocino Pass. We also observed non-significant differences in 11 pairwise comparisons (out of 28).

Supplementary Tables and Figures

Table A: **Sample sizes for *Neophasia menapia* for wing melanization and wing shape.**

**Location**	**Melanization**	**Wing Shape**
Donner Pass (DP)	25	23
Lang (LA)	14	14
Woodfords (WO)	30	29
Goat Mountain Early (GE)	30	40
Goat Mountain Late (GL)	31	42
Mendocino Pass Early (ME)	37	40
Mendocino Pass Late (ML)	18	20
Oregon (OR)	11	14

Table B: Results of Tukey's HSD test for wing melanization. Significant differences are highlighted in bold.

	DP	LA	WO	GE	GL	ME	ML	OR
DP								
LA	-4.691							
WO	**-25.606**	**-20.915**						
GE	**-30.576**	**25.885**	4.970					
GL	0.097	-4.788	**-25.703**	**-30.673**				
ME	**-31.932**	**-27.241**	6.326	-1.356	**-32.029**			
ML	**-11.307**	-6.616	**-14.296**	**19.268**	**-11.405**	**-20.624**		
OR	**-21.093**	**-16.402**	-4.513	9.483	**-21.190**	10.839	-9.785	

Table C: Pairwise Procrustes distances among populations for shape (upper triangle), and melanization level (lower triangle). *P≤0.05, **P≤0.01, ****P≤0.001.**

	DP	LA	WO	GE	GL	ME	ML	OR
DP		**0.029****	0.027**	**0.032****	**0.022****	**0.027****	0.018	0.024*
LA	5.79		0.026**	**0.041****	**0.035****	0.023**	0.026**	**0.041****
WO	**27.47****	**21.68****		0.017*	**0.024****	0.018**	**0.027****	**0.039****
GE	**31.05****	**25.26****	3.57		**0.025****	**0.025****	**0.036****	**0.039****
GL	3.87	9.66	**31.34****	**34.92****		**0.026****	0.021**	0.026**
ME	**33.33****	**27.54****	5.85	5.85	**37.20****		**0.027****	**0.036****
ML	10.60	4.81	16.87**	**20.44****	14.47*	**22.73****		**0.03****
OR	15.00*	9.21	12.47	16.04*	18.87**	18.33**	4.39	

Table D: One-way ANOVA of PC1 by sampling location.

Source of Variation	Degrees of Freedom	Sums of Squares	Mean Squares	F Ratio	P
Population	7	0.02119	0.0030278	7.687	2.78e-08
Residuals	214	0.08429	0.0003939		

Table E: One-way ANOVA of PC2 by sampling location.

Source of Variation	Degrees of Freedom	Sums of Squares	Mean Squares	F Ratio	P
Population	7	0.01379	0.0019703	7.959	1.39e-08
Residuals	214	0.05298	0.0002476		

Table F: One-way ANOVA of PC3 by sampling location.

Source of Variation	Degrees of Freedom	Sums of Squares	Mean Squares	F Ratio	P
Population	7	0.01602	0.0022890	11.32	3.42e-08
Residuals	214	0.04326	0.0002021		

Table G: Results of Tukey's HSD test for PC1. Significant differences are highlighted in bold.

	DP	LA	WO	GE	GL	ME	ML	OR
DP								
LA	1.140e-02							
WO	-1.384e-02	**-2.524e-02**						
GE	**-2.234e-02**	**3.374e-02**	8.498e-03					
GL	-4.949e-03	1.635e-02	-8.893e-03	**1.739e-02**				
ME	-3.243e-03	-1.465e-02	-1.060e-02	**1.910e-02**	1.705e-03			
ML	6.705e-03	-4.697e-03	**-2.055e-02**	**2.904e-02**	1.165e-02	9.948e-03		
OR	-3.341e-03	-1.474e-02	-1.050e-02	**1.900e-02**	1.607e-03	-9.849e-05	-1.005e-02	

Table H: Results of Tukey's HSD test for PC2. Significant differences are highlighted in bold.

	DP	LA	WO	GE	GL	ME	ML	OR
DP								
LA	-0.009							
WO	-0.011	-0.002						
GE	**-0.018**	0.009	0.007					
GL	-0.006	-0.004	-0.006	**0.013**				
ME	**-0.020**	-0.011	0.009	-0.002	**-0.015**			
ML	0.001	-0.010	-0.012	**0.019**	0.006	**0.021**		
OR	-0.001	0.008	-0.010	**0.017**	0.004	**0.019**	-0.002	

Table I: Results of Tukey's HSD test for PC3. Significant differences are highlighted in bold.

	DP	LA	WO	GE	GL	ME	ML	OR
DP								
LA	**0.021**							
WO	**0.017**	-0.004						
GE	0.007	**0.014**	0.010					
GL	0.000	**0.021**	**0.017**	-0.007				
ME	**0.014**	-0.008	0.003	0.006	**0.014**			
ML	0.006	**-0.015**	0.011	-0.001	0.006	-0.008		
OR	-0.012	**-0.034**	**0.029**	**-0.019**	-0.012	**-0.026**	**-0.018**	

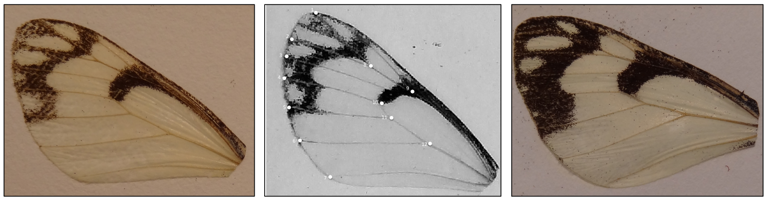

Figure A: **Location of Landmarks.** Left panel: male forewing from Goat Mountain early flight. Middle panel: location of 12 landmarks on *N. menapia* forewing, wing changed to greyscale in ImageJ. Right panel: Male forewing from Goat Mountain late flight.

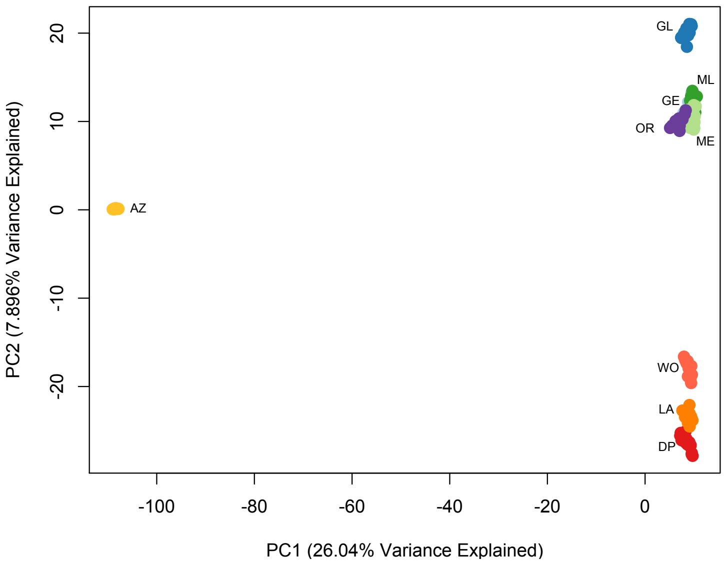

Figure B: **Principal Component Analysis of Genotype Posterior Probabilities.** PCA based on genotype posterior probabilities where each circle represents an individual's genotype posterior probabilities across all 20,737 SNPs; PCA for *N. terlootii* and *N. menapia*. AZ (yellow) = *N. terlootii* from Arizona, *N. menapia* samples from; DP (red) = Donner Pass, LA (orange) = Lang, WO (light red) = Woodfords, GE (light blue) = Goat Mountain early flight, GL (dark blue) = Goat Mountain late flight, ME (light green) = Mendocino Pass early flight, ML (dark green) = Mendocino Pass late flight, OR (purple) = Oregon.

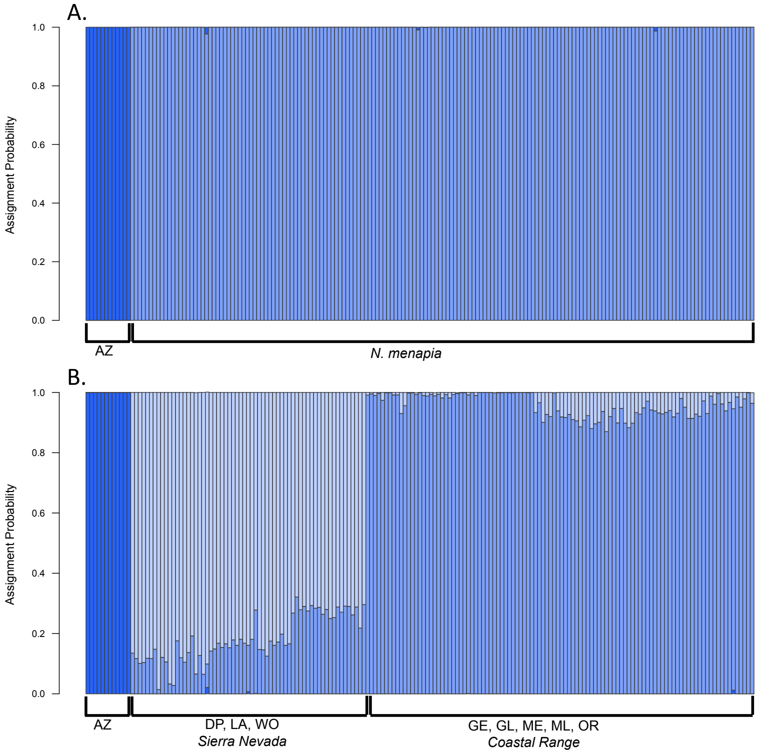

Figure C: **Structure Plot for All Populations.** A: STRUCTURE assignment plot for K=2, includes all populations samples (*N. terlootii* and *N. menapia*); dark blue = AZ (*N. terlootii*), medium blue = all *N. menapia* populations. B: STRUCTURE assignment plot for K=3, includes all populations samples (*N. terlootii* and *N. menapia*), dark blue = AZ, light blue = Sierra Nevada *N. menapia*, medium blue = Coast Range *N. menpia*. AZ = *N. terlootii*, DP= Donner Pass, GE = Goat Mountain early flight, GL = Goat Mountain late flight, LA = Lang, ME = Mendocino Pass early flight, ML = Mendocino Pass late flight, OR = Oregon, WO = Woodfords.

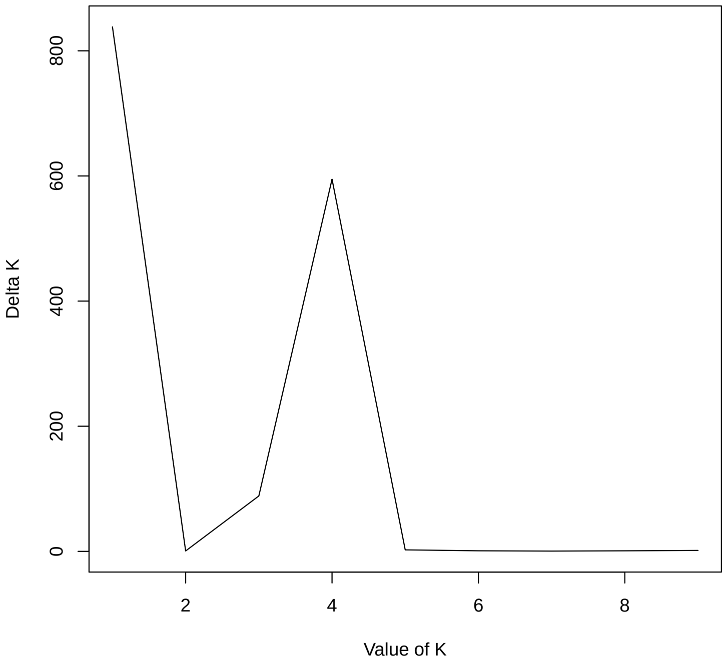

Figure D: **Delta K for K 2 through K 10 for *N. menapia* STRUCTURE Runs**

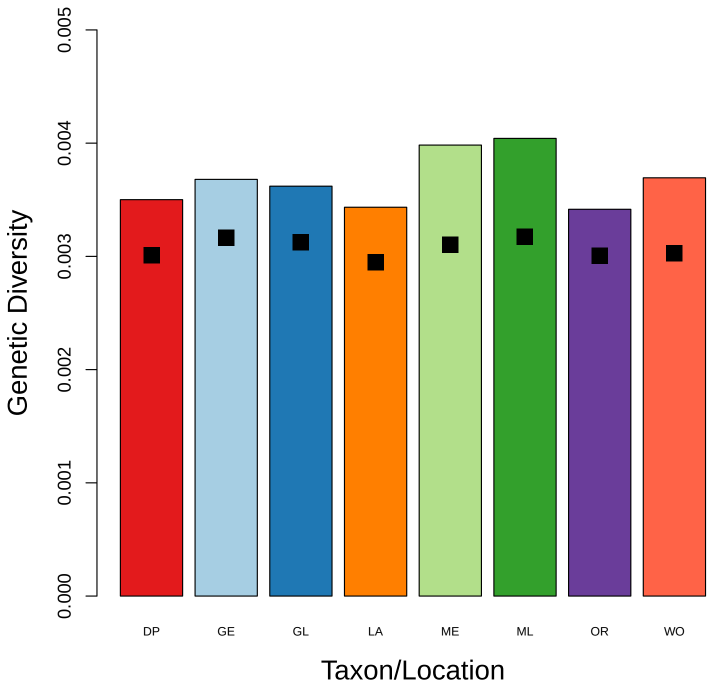

Figure E: **Genetic Diversity Estimates for *N. menapia*** Bars show estimates of heterozygosity (π), square shows the estimate of Watterson's θ.

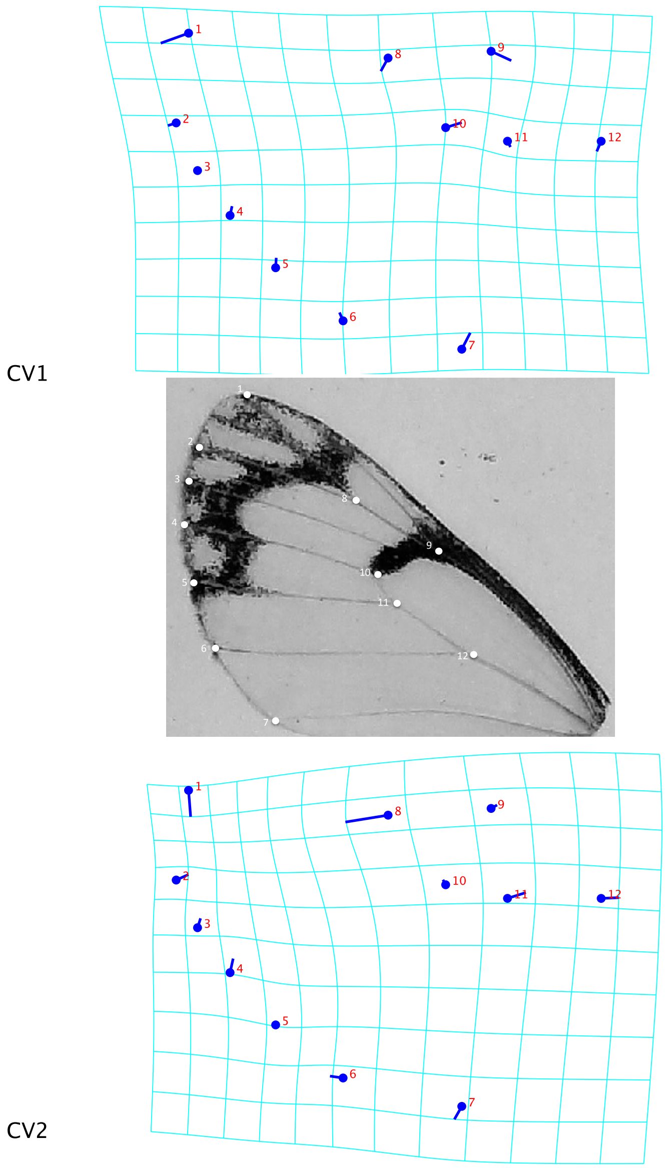

Figure F: **Transformation Grid for Landmarks.** Transformation grid for landmarks from CV1 (top) and CV2 (lower).

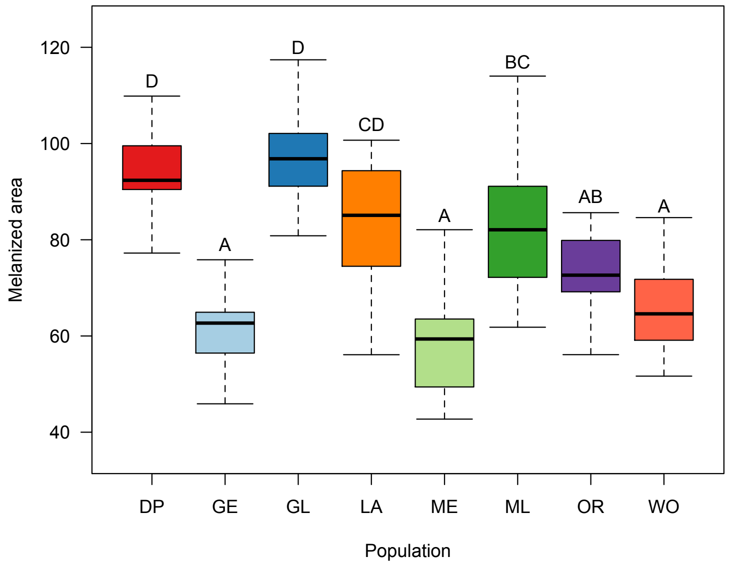

Figure G: Boxplots of melanization level for populations of *Neophasia menapia*. Unique letters indicate significant differences in melanization (calculated by Procrusted distance ANOVA. DP = Donner Pass, GE = Goat Mountain early flight, GL = goat Mountain late flight, LA = Lang, ME = Mendocino Pass early flight, ML = Mendocino Pass late flight, OR = Oregon, WO = Woodfords.

References

Adams, D.C. & Otárola-Castillo, E. 2013. Geomorph: an R package for the collection and analysis of geometric morphometric shape data. *Methods Ecol. Evol.* **4**: 393–399.

Collyer, M., Sekora, D. & Adams, D. 2014. A method for analysis of phenotypic change for phenotypes described by high-dimensional data. *Heredity* .

Rohlf, F.J. & Slice, D. 1990. Extensions of the procrustes method for the optimal superimposition of landmarks. *Syst. Biol.* **39**: 40–59.

Schneider, C.A., Rasband, W.S. & Eliceiri, K.W. 2012. NIH Image to ImageJ: 25 years of image analysis. *Nat. Methods* **9**: 671–675.
